# Age Dependent Epidemic Modeling of COVID-19 Outbreak in Kuwait, France, and Cameroon

**DOI:** 10.3390/healthcare10030482

**Published:** 2022-03-04

**Authors:** Kayode Oshinubi, Sana S. Buhamra, Noriah M. Al-Kandari, Jules Waku, Mustapha Rachdi, Jacques Demongeot

**Affiliations:** 1 Laboratory AGEIS EA 7407, Team Tools for e-Gnosis Medical, Faculty of Medicine, University Grenoble Alpes (UGA), 38700 La Tronche, France; kayode.oshinubi@univ-grenoble-alpes.fr (K.O.); mustapha.rachdi@univ-grenoble-alpes.fr (M.R.); jacques.demongeot@univ-grenoble-alpes.fr (J.D.); 2 Department of Information Science, Kuwait University, P.O. Box 5969, Safat 13060, Kuwait; 3 Department of Statistics and Operations Research, Kuwait University, P.O. Box 5969, Safat 13060, Kuwait; noriah.alkandari@ku.edu.kw; 4 UMMISCO UMI IRD 209 & LIRIMA, University of Yaoundé I, Yaoundé P.O. Box 337, Cameroon; jules.waku@facsciences-uy1.cm

**Keywords:** SIR model, COVID-19, age-dependent modeling, demographic model, epidemic model

## Abstract

Revisiting the classical model by Ross and Kermack-McKendrick, the Susceptible–Infectious–Recovered (SIR) model used to formalize the COVID-19 epidemic, requires improvements which will be the subject of this article. The heterogeneity in the age of the populations concerned leads to considering models in age groups with specific susceptibilities, which makes the prediction problem more difficult. Basically, there are three age groups of interest which are, respectively, 0–19 years, 20–64 years, and >64 years, but in this article, we only consider two (20–64 years and >64 years) age groups because the group 0–19 years is widely seen as being less infected by the virus since this age group had a low infection rate throughout the pandemic era of this study, especially the countries under consideration. In this article, we proposed a new mathematical age-dependent (Susceptible–Infectious–Goneanewsusceptible–Recovered (SIGR)) model for the COVID-19 outbreak and performed some mathematical analyses by showing the positivity, boundedness, stability, existence, and uniqueness of the solution. We performed numerical simulations of the model with parameters from Kuwait, France, and Cameroon. We discuss the role of these different parameters used in the model; namely, vaccination on the epidemic dynamics. We open a new perspective of improving an age-dependent model and its application to observed data and parameters.

## 1. Introduction

### 1.1. Background

**Figure 1:**
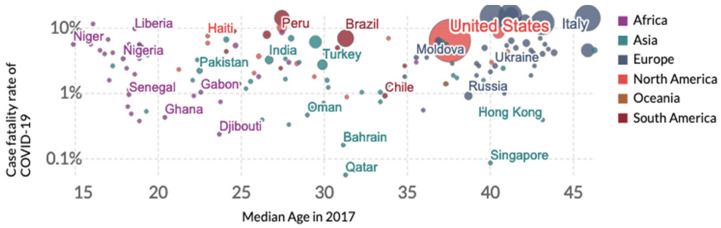
Dependence of the case fatality rate (from cumulative deaths on the 20 May 2020) vs. median age of several countries in 2017 (from [1]). The area of a country circle is proportional to the number of cumulated deaths due to COVID-19 on the 20 May 2020, e.g., for the USA: 99,643 (in red).

**Figure 2:**
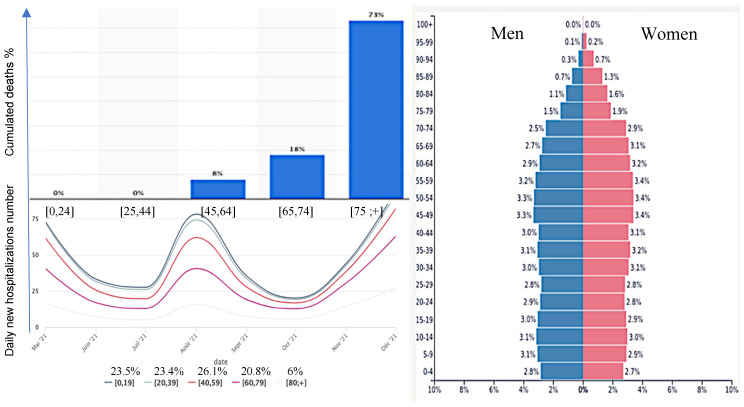
**Top left**: COVID-19 percentage of death in France by age class [23]. **Bottom left**: Influence of age (curves with color coding) on COVID-19 hospitalizations in France in the extreme age classes [23]. **Bottom right**: age classes pyramid in 2020 in France (total population size: 65,273,512) [24].

The influence of the median age of a country on the incidence of COVID-19 has been highlighted on numerous occasions, in particular through the appearance of many asymptomatic cases in the age groups below 50. For example, on the site of Johns Hopkins University dedicated to COVID-19 [1], data clearly shows this influence on the case fatality rate corresponding to the cumulative deaths recorded 5 months after the beginning of the outbreak (12 May 2020) vs. the median age of many countries in 2017 (Figure 1). This first observation has been confirmed by many studies in different countries [2,3,4,5,6,7,8,9,10,11,12,13,14,15,16,17,18,19,20,21,22] such as in France (Figure 2), and we will confirm in this paper that the age of the patients suffering from COVID-19 is a good predictor of severity.

This severity among different age groups increased COVID-19 incidence at the start of the pandemic in terms of hospitalization cases, intensive care unit (ICU) cases, prompting many countries to quickly implement intervention measures such as increasing the number of beds and ventilators in their various hospitals and setting up new isolation centers in order to mitigate its spread and save lives, particularly the elderly, who were disproportionately among those who were highly susceptible.

### 1.2. Related Work

The heterogeneity in the age of the populations studied here (Kuwait, France, and Cameroon) leads to considering a model in age groups with specific susceptibilities for each age class, which makes the prediction problem of the new infectious growth more difficult. Basically, there are three age groups of interest in the COVID-19 outbreak, which are, respectively, 0–19 years, 20–64 years, and >64 years, but here we only consider two (20–64 years and >64 years) age groups because the group 0–19 years is widely seen as being less infectious by SARS-CoV-2 [21,22] since this age group had a low infection rate throughout the period considered in this study, especially the countries data used for simulation.

The heterogeneity in individual age and the reaction to biological and environmental changes that have been observed in COVID-19 dynamics in terms of different reactions to vaccination by age group, severity of infection per age group, hospitalization, and intensive care unit (ICU) records show different patterns, which is why it is important to improve mathematical models for COVID-19 pandemic prediction to account for different proportions of ages in the population, which is a major factor in epidemic history [25]. In some age classes (namely young and adult classes), all infectious are neither symptomatic nor reported and some models incorporating asymptomatic [26], unreported [27], and super-spreaders [28] have been proposed for the COVID-19 outbreak. Many authors have also included age in their models as a gravity factor [29,30,31,32,33], but here, the originality is to propose an improvement of the classical Ross and Kermack-McKendrick model and use it for giving a theoretical and numerical framework for interpreting the relationship between demographic parameters such as age pyramid, fertility and mortality, and epidemiological parameters such as the basic reproduction number R_0_ and vaccination rate.

In subsequent sections, we present (i) in Section 2, the materials and methods, (ii) in Section 3, the formulation of a new age-dependent dynamical model for the spread of COVID-19, (ii) in Section 4, we provide some mathematical analyses of the model, (iii) in Section 5, we carry out numerical simulations for some COVID-19 parameters from Kuwait, France, and Cameroon; lastly (iv), in Section 6 and Section 7, we provide a discussion and some conclusions.

## 2. Material and Methods

### 2.1. Material

**Figure 3:**
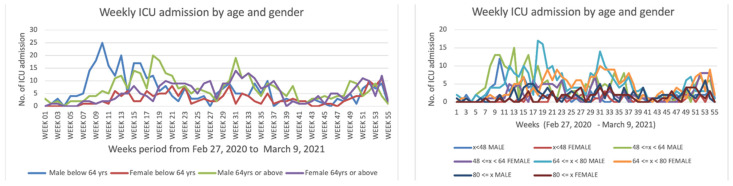
Curves of COVID-19 weekly ICU admissions in Kuwait by age classes (two on the **left** and four on the **right**) and gender from 27 February 2020 to 9 March 2021.

**Figure 4:**
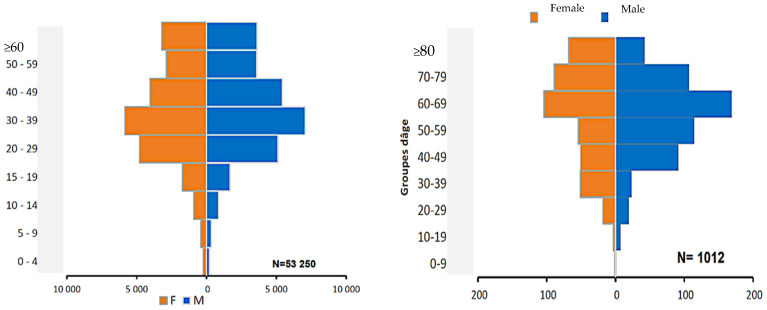
**Left**: Distribution of cumulated confirmed cases of COVID-19 by age group and gender in Cameroon as of 23 June 2021 [25]. **Right**: Distribution of deaths due to COVID-19 infection by age group and gender in Cameroon as of 23 June 2021 (after [34]).

The data material is from public epidemiologic and demographic databases [23,24] and the reference methods are both from classical demographic (such as Leslie) and epidemiologic (such as Ross and Kermack-McKendrick) models. In Figure 3 and Figure 4, we present visualizations for pandemic dynamics in different age groups and sexes for Kuwait and Cameroon, respectively, to support the motivation for this article that pandemic evolution and severity are related to age classes, and thus modeling is important as various researchers and health experts are investigating SAR-CoV-2 mutations. The countries under consideration have a higher proportion of young people, while the elderly have a lower proportion. Only 2% of the total population in Kuwait is over 65 and vulnerable to the pandemic, whereas 20.8% of the population in France is over 65 and vulnerable to the pandemic, and 2.7% of the population in Cameroon is over 65 and vulnerable to the pandemic.

### 2.2. Leslie Model

The first population growth model was proposed by Leslie in 1945 using the age pyramid vector $p\left(t\right)={\left(p_{j}\left(t\right)\right)}_{j=1,2,\ldots ,m},$ where $p_{j}\left(t\right)$ represents the size of the age class j at time t, with j ranging from the birth age 1 to the maximal death age m, whose discrete dynamics is governed by the matrix equation given as:

$$p\left(t\right)=\mathrm{Lp}\left(t-1\right)\;\mathrm{with}\;L=\left(l_{\mathrm{jk}}\right)=\left[\begin{array}{ccccc} f_{1} & f_{2} & f_{3} & \ldots & \ldots \\ b_{1} & 0 & 0 & \ldots & \ldots \\ 0 & b_{2} & 0 & \ldots & \ldots \\ \vdots & \vdots & \ddots & \ldots & \ldots \\ \vdots & \vdots & \vdots & \ddots & \ldots \\ 0 & 0 & 0 & \ldots & b_{m-1} \end{array}\begin{array}{c} f_{m}\\ 0\\ 0\;\\ \vdots \\ \vdots \\ \;0 \end{array}\right]$$

where ∀ j = 1, …, m, b_j_ = 1 − μ_j_ ≤ 1 (where µ*_j_* is the death rate at age j) is the survival probability between ages j and j + 1 and $f_{1}$ is the birth rate at age j (i.e., the mean number of offsprings from an individual at age j). The dynamic stability of the Leslie system for a distance d is quantified by the tendency to return to its stationary pyramid p* after a perturbation p* + q, such as d(p*,L^m^(p* + q)) < Ke^−mD^ tends exponentially to 0, when m goes to infinity, the parameter D being the stability module. For the $L^{2}$ distance to the stationary pyramid p*, D = |λ-λ’|, absolute value of the difference between the dominant and sub-dominant eigenvalues of L, i.e., λ and λ’ (λ = e^r^, where r is the Malthusian growth rate, and p* is the left eigenvector of L corresponding to λ). For the distance (known as symmetrized divergence) of Kullback–Leibler, D = kH, where H is the entropy of p* and k is a constant [35,36,37,38,39,40,41,42,43,44,45,46].

### 2.3. Volterra Integral

The Volterra integral equation of the second kind is given as:

$$S\left(t\right)=\;S\left(0\right)+\int_{0}^{t}H\left(t,\epsilon \right)S\left(\epsilon \right)d\epsilon$$

where $H\left(t,\epsilon \right)$ is a kernel and $S\left(t\right)$ is the function to be solved.

### 2.4. The Ross-McKendrick SIR Model

Bernoulli [47] proposed a model for explaining the smallpox dynamics and since then, many discussions have occurred regarding the efficacy of firstly the inoculation and secondly the vaccination [48,49,50,51]. In Bernoulli’s model, the population is divided into susceptibles and immunized; the probability of belonging to them is denoted as u and v. The probability for a newborn individual susceptible and alive at age j and the probability of being immunized and alive at age j is denoted as ν_j_. Ross [52] and McKendrick [53] (assisted later by W.O. Kermack) proposed an improvement of Bernoulli’s model called the SIR model with the following equations:

$$\frac{\mathrm{dS}}{\mathrm{dt}}=\delta S+\delta I+\left(\delta +\gamma \right)R-\beta \mathrm{SI}-µS,\frac{\mathrm{dI}}{\mathrm{dt}}=\beta \mathrm{SI}-\left(µ+\nu \right)I,\;\frac{\mathrm{dR}}{\mathrm{dt}}=\nu I-\left(µ+\gamma \right)R$$

where S is the size of the susceptible, I the size of the infectious, and R the size of the recovered with the total population N defined as N = S + I + R. β is the contagion rate, δ the birth rate (supposed to be equal to the death rate), γ is the loss of immune resistance, and ν is the immunization rate. The basic reproduction number $R_{0}=\frac{\beta N}{\nu +\delta }$ is the mean number of individuals secondarily infectious by one primary infectious. $R_{0}$ predicts, if it is greater than 1, the occurrence of an epidemic wave. By defining age classes between 1 and m and by denoting $S_{j},{\;I}_{j},R_{j},\;j=1,\;2,\ldots ,m,$ each subpopulation of S, I, and R at age j, we can define at any stationary state ($S^{*},I^{*},R^{*}$) the probabilities for a newborn individual of being alive and either susceptible, infectious or immune at age j by the following formula:

$$u^{*}\left(j\right)=\frac{S_{j}{}^{*}}{P^{*}},{\;v}^{*}\left(j\right)=\frac{I_{j}{}^{*}}{P^{*}},{\;w}^{*}\left(j\right)=\frac{R_{j}{}^{*}}{P^{*}},\;\mathrm{where}\;P*=\Sigma_{i=1,m}\;(S_{i}+I_{i}+R_{i})$$

That makes the link between the Bernoulli and the Ross and Kermack-McKendrick models, but the weakness of the latter still resides in many insufficiencies and approximations:

- When the population size of either susceptible or infectious populations tends to be very large, the quadratic term SI has to be replaced by a Michealian saturation term $\frac{\mathrm{SI}}{\left(k+S\right)\left(\;k\;\prime +I\right)}$
- the immunized infectious or healthy carriers are neglected in the absence of real information on their quantity and their influence on the spread of COVID-19;
- the total population size is supposed to be constant, the birth rate equaling the natural mortality. The Bernoulli model [47] takes implicitly into account the birth rate, and explicitly the natural mortality. The model by d’Alembert [54,55] improved Bernoulli’s model by distinguishing the specific mortality due to the infectious disease from the natural one, being more widely applicable than the model by Bernoulli which was restricted to immunizing infections. In the d’Alembert method, the only task was to calculate the survival function after eliminating the particular cause of death due to the infectious disease, but Bernoulli’s approach provided much more insight for a mechanistic interpretation of infectious disease real data;
- variables and parameters do not depend on space, i.e., neither migration nor population displacement;
- parameters do not depend on time which means no genetic adaptation of an infectious agent or a human population, even if it is very slow compared to the fast dynamics of epidemics.

Our proposed model is an improvement of the Ross and Kermack-McKendrick model by trying to compensate for a part of these defects. We will first introduce two age classes to account for adults and the elderly in the population and then take account of vaccination before applying our model to some countries chosen as examples.

## 3. New SIGR Model Formulation

We propose a Susceptible–Infectious–Goneanewsusceptible–Recovered (SIGR) model as an improvement of the Ross and Kermack-McKendrick models including age class and vaccination state for COVID-19 in a given population. Neglecting differences between kids and young adults, we only retain two age classes: adult and elderly, 1 and 2. We assume that all infectious are symptomatic and we consider birth and natural death rates ß and µ, as well as specific fatality rate ϵ due to the disease. Age groups (i = 1,2) concern individuals susceptible, infectious $I_{i},$ gone anew susceptible G_i_, and a fully recovered and resistant R_i_. We denote for each age group (i = 1,2) the transmission rates $\varphi_{i\;}$, fertility, and loss of resistance rates β_ij_ (supposed to be equal inside an age class, for the sake of simplicity), natural death rates $\mu_{2}{}^{S},{\;\mu }_{2}{}^{I},{\;\mu }_{2}{}^{G}{\;\mathrm{and}\;\mu }_{2}{}^{R}$, vaccination rates group $\theta_{i}^{G}$ and $\theta_{i}^{R},$ survival rates from age 1 to age 2 $a_{1}^{S},{\;a}_{1}^{I},{\;a}_{1}^{G}{\;\mathrm{and}\;a}_{1}^{R},\;$ specific death rate due to the disease $\epsilon_{i\;},\;$ relapsed rate ${\;\eta }_{i}$ and recovery rate $\gamma_{i}\;$(cf. Table 1).

**Figure 5:**
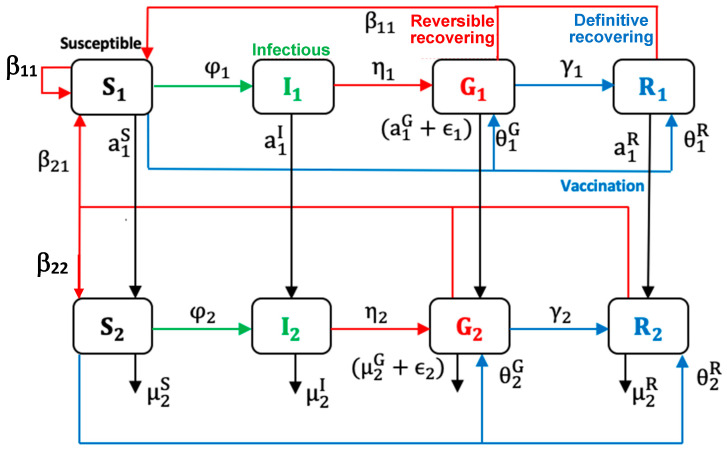
Age-dependent scheme for COVID-19 outbreak modeling.

The description above can be illustrated by the following set of non-linear differential Equation (1), while the graphical representation of the model is given in Figure 5:

(1) $${\mathrm{dS}}_{1}/\mathrm{dt}=\beta_{11}\;(S_{1}+G_{1}+R_{1})+\beta_{21}(S_{2}+G_{2}+R_{2})\;–\;(a_{1}^{S}+\theta_{1}^{G}+{\;\theta }_{1}^{R}+\varphi_{1}(I_{1}\;+I_{2}))S_{1}\;{\mathrm{dS}}_{2}/\mathrm{dt}=a_{1}^{S}S_{1}\;–\;\mu_{2}{}^{S}S_{2}\;-\;(\theta_{2}^{G}+{\;\theta }_{2}^{R}+\varphi_{2}(I_{1}+I_{2}))S2_{\;}+\beta_{22}(G_{2}+R_{2})\;{\mathrm{dI}}_{1}/\mathrm{dt}=\varphi_{1}\left(I_{1}+I_{2}\right)S_{1}-(a_{1}^{I}+\eta_{1})I_{1}\;{\mathrm{dI}}_{2}/\mathrm{dt}=\varphi_{2}\left(I_{1}+I_{2}\right)S_{2}+a_{1}^{I}I_{1}-\eta_{2}{\;I}_{2}\;–\;\mu_{2}{}^{I}I_{2}\;{\mathrm{dG}}_{1}/\mathrm{dt}={\;\eta }_{1}I_{1}+{\;\theta }_{1}^{G}S_{1}\;–\;(a_{1}^{G}+\gamma_{1}+\epsilon_{1})G_{1}\;\;{\mathrm{dG}}_{2}/\mathrm{dt}={\;\eta }_{2}I_{2}+{\;a}_{1}^{G}G_{1}+{\;\theta }_{2}^{G}S_{2}\;–\;(\gamma_{2}+{\;\mu }_{2}{}^{G}+\epsilon_{2}+\beta_{22})G_{2}\;{\mathrm{dR}}_{1}/\mathrm{dt}=\gamma_{1}G_{1}+\theta_{1}^{R}S_{1}-{\;a}_{1}^{R}R_{1}\;{\mathrm{dR}}_{2}/\mathrm{dt}=\gamma_{2}G_{2}+\theta_{2}^{R}S_{2}+a_{1}^{R}R_{1}\;–\;(\mu_{2}{}^{R}+\beta_{22})R_{2}$$

with $\;S_{1}\left(t\right)\geq 0,S_{2}\left(t\right)\geq 0,{\;I}_{1}\left(t\right)\geq 0,{\;I}_{2}\left(t\right)\geq 0,{\;G}_{1}\left(t\right)\geq 0,{\;G}_{2}\left(t\right)\geq 0,{\;R}_{1}\left(t\right)\geq 0,{\;R}_{2}\left(t\right)\geq 0.$

We will suppose in the following that the loss of resistance rates is neglectable.

## 4. Mathematical Analysis of the Model

### 4.1. Positivity of the Solution

**Lemma** **1.** *Let the initial condition be given as follows:*

$\left\{S_{1}\left(0\right),S_{2}\left(0\right),{\;I}_{1}\left(0\right),{\;I}_{2}\left(0\right),{\;G}_{1}\left(0\right),{\;G}_{2}\left(0\right),{\;R}_{1}\left(0\right),{\;R}_{2}\left(0\right)\geq 0\right\},\;$ *then solutions of system of*

*Equation (1) are positive for all* t>0.

**Proof**  From the first equation in the model Equation (1), we obtain:

$${\mathrm{dS}}_{1}/\mathrm{dt}=\beta_{11}\;(S_{1}+G_{1}+R_{1})+\beta_{21}\;(S_{2}+G_{2}+R_{2})\;–\;(a_{1}^{S}+\theta_{1}^{G}+{\;\theta }_{1}^{R}+\varphi_{1}(I_{1}+I_{2}))S_{1},$$

Hence, dS_1_/dt $\geq (\beta_{11\;}-a_{1}^{S}-\theta_{1}^{G}-\theta_{1}^{R}$) S_1_

By using the separating variable method and then, integrating, we obtain:

$$\int \frac{{\mathrm{dS}}_{1}/\mathrm{dt}}{S_{1}}\;\geq \int \left(\beta_{11\;}-a_{1}^{S}-\theta_{1}^{G}-\theta_{1}^{R}\right)\mathrm{dt}$$

$$\ln S_{1}\geq \left(\beta_{11\;}-a_{1}^{S}-\theta_{1}^{G}-\theta_{1}^{R}\right)t+k$$

Finally, by writing S_1_(0) = e^k^, we have: $S_{1}\left(t\right)\geq S_{1}\left(0\right)e^{\left(\beta_{11}-a_{1}^{S}-\theta_{1}^{G}-\theta_{1}^{R}\right)t}\geq 0$.

By applying the same process to other equations in (1), we have:

$$S_{2}\left(t\right)\geq S_{2}\left(0\right)e^{\left(a_{1}^{S}-\theta_{2}^{G}-\theta_{2}^{R}-\mu_{2}{}^{S}\right)t}\geq 0$$

$$I_{1}\left(t\right)\geq I_{1}\left(0\right)e^{-\left(a_{1}^{I}+\eta_{1}\right)t}\geq 0$$

$$I_{2}\left(t\right)\geq I_{2}\left(0\right)e^{-\left(\eta_{2}{\;I}_{2}+{\;\mu }_{2}{}^{I}\right)t}\geq 0$$

$$G_{1}\left(t\right)\geq G_{1}\left(0\right)e^{-\left(a_{1}^{G}+\gamma_{1}+\epsilon_{1}\right)t}\geq 0$$

$$G_{2}\left(t\right)\geq G_{2}\left(0\right)e^{-\left(\gamma_{2}+{\;\mu }_{2}{}^{G}+\epsilon_{2}\right)t}\geq 0$$

$$R_{1}\left(t\right)\geq R_{1}\left(0\right)e^{-a_{1}^{R}t}\geq 0$$

$$R_{2}\left(t\right)\geq R_{2}\left(0\right)e^{-\mu_{2}{}^{R}t}\geq 0$$

Then, the solutions of Equation (1) $\left\{S_{1}\left(t\right),S_{2}\left(t\right),{\;I}_{1}\left(t\right),{\;I}_{2}\left(t\right),{\;G}_{1}\left(t\right),{\;G}_{2}\left(t\right),{\;R}_{1}\left(t\right),{\;R}_{2}\left(t\right)\right\}$ are positive for all t>0.  □

### 4.2. Boundedness of the Solution

Let denote by S the total size of all individuals:

S(t) = S_1_(t) + S_2_(t) + I_1_(t) + I_2_(t) + G_1_(t) + G_2_(t) + R_1_(t) + R_2_(t)

Then, by adding all the model Equations (1), we have:

$$\mathrm{dS}/\mathrm{dt}=\beta_{11}\;(S_{1}+G_{1}+R_{1})+\beta_{21}\;(S_{2}+G_{2}+R_{2})\;–\;({{µ}_{2}}^{S}S_{2}+{{µ}_{2}}^{I}I_{2}+{{µ}_{2}}^{G}{G_{2}}^{\;}+\epsilon G_{2}+{{µ}_{2}}^{R}{R_{2}}^{}$$

Let us denote µ = inf{µ_2_^S^, µ_2_^I^, µ_2_^G^+𝜖, µ_2_^R^}. By neglecting the fecundity rate of the young class and if β = β_21_ ≤ µ, we have:

dS/dt ≤ β(S_2_ + I_2_ + G_2_ + R_2_) – µ(S_2_ + I_2_ + G_2_ + R_2_) ≤ 0,

Then, we can conclude that the total size S is bounded, which implies the boundedness of the partial sizes $S_{1}\left(t\right),S_{2}\left(t\right),{\;I}_{1}\left(t\right),{\;I}_{2}\left(t\right),{\;G}_{1}\left(t\right),{\;G}_{2}\left(t\right),{\;R}_{1}\left(t\right),{\;R}_{2}\left(t\right)$.

### 4.3. Disease-Free (Eradication) Equilibrium

Setting the right hand side of the model equations (1) to zero, i.e.,

$${\mathrm{dS}}_{1}/\mathrm{dt}={\mathrm{dS}}_{2}/\mathrm{dt}={\mathrm{dI}}_{1}/\mathrm{dt}={\mathrm{dI}}_{2}/\mathrm{dt}=G_{1}/\mathrm{dt}={\mathrm{dG}}_{2}/\mathrm{dt}={\mathrm{dR}}_{1}/\mathrm{dt}={\mathrm{dR}}_{2}/\mathrm{dt}=0$$

and supposing that all infectious class sizes are equal to zero which means that there is no disease eradication in the studied population, thus the disease-free equilibrium state is:

$$(S_{1}{}^{*},{\;S}_{2}{}^{*},I_{1}{}^{*},I_{2}{}^{*}G_{1}{}^{*},G_{2}{}^{*},R_{1}{}^{*},R_{2}{}^{*})=\left\{\frac{\beta_{21\;}S_{2}}{a_{1}^{S}+\theta_{1}^{G}+{\;\theta }_{1}^{R}-\beta_{11\;}},\frac{a_{1}^{S}S_{1}}{\mu_{2}{}^{S}+\theta_{2}^{G}+{\;\theta }_{2}^{R}},0,0,0,0,0,0\right\}$$

### 4.4. Proof of Stability of the Endemic State

Let us fix a set of values for the model parameters, where k is a scale parameter:

$$\varphi_{1\;}={\;\varphi }_{2\;}=\frac{4k^{2}}{100},{\;\mu }_{2}{}^{S}=\epsilon_{1\;}=\beta_{22\;}=0,{\;\mu }_{2}{}^{I}=\frac{499k}{100},{\;\mu }_{2}{}^{G}=\frac{49k}{100},{\;\mu }_{2}{}^{R}=\frac{2k}{5},{\;\beta }_{11\;}={\;\beta }_{21\;}=\frac{k}{5},$$

$$\theta_{1}^{G}=\theta_{1}^{R}=\gamma_{1}=\gamma_{2}=0.1k,{\;\theta }_{2}^{G}=\theta_{2}^{R}=k,{\;a}_{1}^{S}={\;a}_{1}^{I}={\;a}_{1}^{G}={\;a}_{1}^{R}=\frac{98k}{96},\;\epsilon_{2\;}=\frac{4k}{5},{\;\eta }_{1}={\;\eta }_{2}=0.2k$$

The two stationary points are labeled with * (resp. **) for the eradication (resp. endemic) state:

$$(S_{1}{}^{*},{\;S}_{2}{}^{*},I_{1}{}^{*},I_{2}{}^{*}G_{1}{}^{*},G_{2}{}^{*},R_{1}{}^{*},R_{2}{}^{*})=\left(\frac{\beta_{21\;}S_{2}}{a_{1}^{S}+\theta_{1}^{G}+{\;\theta }_{1}^{R}-\beta_{11\;}},\frac{a_{1}^{S}S_{1}}{\mu_{2}{}^{S}+\theta_{2}^{G}+{\;\theta }_{2}^{R}},0,0,0,0,0,0\right)$$

and

$$(S_{1}{}^{**},{\;S}_{2}{}^{**},I_{1}{}^{**},I^{**},G_{1}{}^{**},G_{2}{}^{**},R_{1}{}^{**},R_{2}{}^{**})=\left(10,10,10,10,20,40,15,15\right),\;\mathrm{if}\;k=1.$$

Let us show that the endemic state is locally stable. With the chosen parameter values and k = 1, from the model Equation (1), we have:

(2) $${\mathrm{dS}}_{1}/\mathrm{dt}=0.2G_{1}-1.02S_{1}+0.2R_{1}+0.2S_{2}+0.2G_{2}+0.2R_{2}-0.04I_{1}S_{1}-0.04I_{2}S_{1}\;{\mathrm{dS}}_{2}/\mathrm{dt}=1.02S_{1}-2S_{2}-0.04I_{1}S_{2}-0.04I_{2}S_{2}\;{\mathrm{dI}}_{1}/\mathrm{dt}=0.04I_{1}S_{1}+0.04I_{2}S_{1}-1.04I_{1}\;{\mathrm{dI}}_{2}/\mathrm{dt}=0.04I_{1}S_{2}+0.04I_{2}S_{2}+1.02I_{1}-5.19I_{2}$$

$${\mathrm{dG}}_{1}/\mathrm{dt}=0.2I_{1}+0.1S_{1}-1.12G_{1}$$

$${\mathrm{dG}}_{2}/\mathrm{dt}=0.2I_{2}+1.02G_{1}+{\;S}_{2}-1.39G_{2}$$

$${\mathrm{dR}}_{1}/\mathrm{dt}=0.1G_{1}+0.1S_{1}-1.02R_{1}$$

$${\mathrm{dR}}_{2}/\mathrm{dt}=0.1G_{2}+S_{2}+1.02R_{1}\;–\;0.4R_{2}$$

By calculating the Jacobian matrix M of the system of Equation (2) at the endemic state, where I is the identity matrix and finding the roots of its characteristic polynomial P_M_, we have for the second stationary point the expression as follows:

$$M-\lambda I=\left(\begin{array}{cccccccc} -1.02-\lambda & 0.2 & -0.4 & -0.4 & 0.2 & 0.2 & 0.2 & 0.2\\ 1.02 & -2-\lambda & -0.4 & -0.4 & 0 & 0 & 0 & 0\\ 0.8 & 0 & -1.04-\lambda & 0 & 0 & 0 & 0 & 0\\ 0 & 0.8 & 1.02 & -5.19-\lambda & 0 & 0 & 0 & 0\\ 0.1 & 0 & 0.2 & 0 & -1.12-\lambda & 0 & 0 & 0\\ 0 & 1 & 0 & 0.2 & 1.02 & -1.39-\lambda & 0 & 0\\ 0.1 & 0 & 0 & 0 & 0.1 & 0 & -1.02-\lambda & 0\\ 0 & 1 & 0 & 0 & 0 & 0.1 & 1.02 & -0.4-\lambda \end{array}\right)$$

The roots of the characteristic polynomial of M, P_M_, satisfy:

$$P_{M}(\lambda )=\det(M-\lambda I)\;=\;\left(\lambda +0.706923)(\lambda +5.13103)(\lambda^{2}+0.973528\lambda +0.34052\right)$$

$$\left(\lambda^{2}+2.0371\lambda +1.03861\right)\left(\lambda^{2}+3.69142\lambda \;+3.53083\right)=0$$

The real parts of the eigenvalues of the matrix M are all negative, equal to:

−0706923, −5.13103, −0.486764, −1.01855, −1.84571

Hence, with the set of chosen parameter values, the stability of the endemic state is proved. 

### 4.5. Existence and Unicity of the Solution

We want to establish existence and unicity of the solution for the model Equation (1). Let us denote the second member of (1) by $H=\left(H_{1},H_{2},H_{3},H_{4},H_{5},H_{6},H_{7},H_{8}\right)$ and the state vector by $Z=(S_{1},S_{2},{\;I}_{1},{\;I}_{2},{\;G}_{1},{\;G}_{2},{\;R}_{1},{\;R}_{2})\;,\;\mathrm{with}\;Z\left(0\right)={\;Z}_{o},\;Z\left(t,\epsilon \right)=H\left(t,\epsilon \right)Z\left(t\right).$ The Volterra integral equation formulation of the model Equation (1) is the following:

(3) $$S_{1}\left(t\right)=S_{1}\left(0\right)+\int_{0}^{t}H_{1}\left(t,\epsilon \right)S_{1}\left(\epsilon \right)d\epsilon \;S_{2}\left(t\right)=S_{2}\left(0\right)+\int_{0}^{t}H_{2}\left(t,\epsilon \right)S_{2}\left(\epsilon \right)d\epsilon \;I_{1}\left(t\right)=I_{1}\left(0\right)+\int_{0}^{t}H_{3}\left(t,\epsilon \right)I_{1}\left(\epsilon \right)d\epsilon \;I_{2}\left(t\right)=I_{2}\left(0\right)+\int_{0}^{t}H_{4}\left(t,\epsilon \right)I_{2}\left(\epsilon \right)d\epsilon \;{\;G}_{1}\left(t\right)=G_{1}\left(0\right)+\int_{0}^{t}H_{5}\left(t,\epsilon \right)G_{1}\left(\epsilon \right)d\epsilon$$

$${\;G}_{2}\left(t\right)=G_{2}\left(0\right)+\int_{0}^{t}H_{6}\left(t,\epsilon \right)G_{2}\left(\epsilon \right)d\epsilon \;R_{1}\left(t\right)=R_{1}\left(0\right)+\int_{0}^{t}H_{7}\left(t,\epsilon \right)R_{1}\left(\epsilon \right)d\epsilon \;R_{2}\left(t\right)=R_{2}\left(0\right)+\int_{0}^{t}H_{8}\left(t,\epsilon \right)R_{2}\left(\epsilon \right)d\epsilon$$

Let $S_{1}$ be the solution for $S_{1}\;\mathrm{and}$ kernels $H_{I},\;i=1,2,\ldots ,\;8$ satisfy Lipschitz conditions: $\sup_{0<t\leq 1}\|S_{1}\|\leq c_{1}$, ${\;\sup}_{0<t\leq 1}\|S_{2}\|\leq c_{2}$, ${\;\sup}_{0<t\leq 1}\|I_{1}\|c_{3},{\;\sup}_{0<t\leq 1}\|I_{2}\|c_{4},{\;\sup}_{0<t\leq 1}\|G_{1}\|\leq c_{5},$ $\sup_{0<t\leq 1}\|G_{2}\|\leq c_{6},{\;\sup}_{0<t\leq 1}\|R_{1}\|\leq c_{7},{\;\sup}_{0<t\leq 1}\|R_{2}\|\leq c_{8\;}{\mathrm{and}\;c}_{i}>0,\;\mathrm{for}\;i=1,\;2,\ldots ,\;8.$ Then, following inequalities hold, using triangle inequality and properties of the $H_{i}^{’}s\;\mathrm{norm}$:

$$\|H_{1}\left(S_{1}\right)-H_{1}\left(S_{1}\right)\|\leq \left[\beta_{11\;}–\;\left(a_{1}^{S}+\theta_{1}^{G}+{\;\theta }_{1}^{R}+{\;\varphi }_{1}\left(I_{1}+I_{2}\right)\right)\right]S_{1}-\left[\beta_{11\;}–\;(a_{1}^{S}+\theta_{1}^{G}+{\;\theta }_{1}^{R}+{\;\varphi }_{1}\left(I_{1}+I_{2}\right))\right]S_{1}\|\;{}_{\;}\;\leq \|\beta_{11\;}–(a_{1}^{S}+\theta_{1}^{G}+{\;\theta }_{1}^{R}+{\;\varphi }_{1}\left(I_{1}+I_{2}\right))\|\|S_{1}-S_{1}\|\;\leq \|\beta_{11\;}–\;(a_{1}^{S}+\theta_{1}^{G}+{\;\theta }_{1}^{R}+{\;\varphi }_{1}c_{3}+\varphi_{1}c_{4})\|\|S_{1}-S_{1}\|\;=\partial_{1}\|S_{1}-S_{1}\|$$

where $\;\xi =\beta_{11\;}$ (G_1_ + R_1_) + β_21_ (S_2_ + G_2_ + R_2_), $\partial_{1}=\beta_{11\;}–{\;a}_{1}^{S}-\theta_{1}^{G}-{\;\theta }_{1}^{R}-{\;\varphi }_{1}c_{3}-\varphi_{1}c_{4}$.

Therefore, $H_{1}$ satisfies the Lipschitz conditions. We can show in the same way that other functions $H_{i},\;i=2,\ldots ,\;8$ in the model Equation (2) satisfy the Lipschitz conditions as follows:

$$\left\|H_{2}\left(S_{2}\right)-H_{2}\left(S_{2}\right)\leq \|\;\partial_{2}\|S_{2}-S_{2}\right\|\;\left\|H_{3}\left(I_{1}\right)-H_{3}\left(L_{1}\right)\leq \;\|\partial_{3}\|I_{1}-L_{1}\right\|\;\left\|H_{4}\left(I_{2}\right)-H_{4}\left(L_{2}\right)\leq \|\;\partial_{4}\|I_{2}-L_{2}\right\|\;\left\|H_{5}\left(G_{1}\right)-H_{5}\left(G_{1}\right)\leq \|\;\partial_{5}\|G_{1}-G_{1}\right\|\;\left\|H_{6}\left(G_{2}\right)-H_{6}\left(G_{2}\right)\leq \|\;\partial_{6}\|G_{2}-G_{2}\right\|\;\left\|H_{7}\left(R_{1}\right)-H_{7}\left({ℜ}_{1}\right)\leq \|\;\partial_{7}\|R_{1}-{ℜ}_{1}\right\|\;\left\|H_{8}\left(R_{2}\right)-H_{8}\left({ℜ}_{2}\right)\leq \|\;\partial_{8}\|R_{2}-{ℜ}_{2}\right\|$$

Let us now consider the following Neumann series:

$$S_{1m}\left(t\right)=S_{1}\left(0\right)+\int_{0}^{t}H_{1}\left(t,\epsilon \right)S_{1m-1}\left(\epsilon \right)d\epsilon \;S_{2m}\left(t\right)=S_{2}\left(0\right)+\int_{0}^{t}H_{2}\left(t,\epsilon \right)S_{2m-1}\left(\epsilon \right)d\epsilon \;I_{1m}\left(t\right)=I_{1}\left(0\right)+\int_{0}^{t}H_{3}\left(t,\epsilon \right)I_{1m-1}\left(\epsilon \right)d\epsilon \;I_{2m}\left(t\right)=I_{2}\left(0\right)+\int_{0}^{t}H_{4}\left(t,\epsilon \right)I_{2m-1}\left(\epsilon \right)d\epsilon \;G_{1m}\left(t\right)=G_{1}\left(0\right)+\int_{0}^{t}H_{5}\left(t,\epsilon \right)G_{1m-1}\left(\epsilon \right)d\epsilon \;G_{2m}\left(t\right)=G_{2}\left(0\right)+\int_{0}^{t}H_{6}\left(t,\epsilon \right)G_{2m-1}\left(\epsilon \right)d\epsilon \;R_{1m}\left(t\right)=R_{1}\left(0\right)+\int_{0}^{t}H_{7}\left(t,\epsilon \right)R_{1m-1}\left(\epsilon \right)d\epsilon \;{\;R}_{2m}\left(t\right)=R_{2}\left(0\right)+\int_{0}^{t}H_{8}\left(t,\epsilon \right)R_{2m-1}\left(\epsilon \right)d\epsilon$$

These Neumann series are convergent due to the Lipschtizian character of H’s, then:

$$\left\|S_{1m+1}-S_{1m}\|\leq \int_{0}^{t}\|H_{1}\left(t,\epsilon \right)S_{1m}\left(\epsilon \right)-H_{1}\left(t,\epsilon \right),S_{1m-1}\left(\epsilon \right)d\epsilon \right\|\;\leq \int_{0}^{t}\|S_{1m}\left(t\right)-S_{1m-1}\left(t\right)\|d\epsilon \;\leq \partial_{1}\|S_{1m}\left(t\right)-S_{1m-1}{\left(t\right)\|}_{\infty }$$

Other equations are given as follows:

$$\|S_{2m+1}-S_{2m}\|\;\leq \partial_{2}\|S_{2m}\left(t\right)-S_{2m-1}{\left(t\right)\|}_{\infty }\;\|I_{1m+1}-I_{1m}\|\;\leq \partial_{3}\|I_{1m}\left(t\right)-I_{1m-1}{\left(t\right)\|}_{\infty }\;\|I_{2m+1}-I_{2m}\|\;\leq \partial_{4}\|I_{2m}\left(t\right)-I_{2m-1}{\left(t\right)\|}_{\infty }\;\|G_{1m+1}-G_{1m}\|\;\leq \partial_{5}\|G_{1m}\left(t\right)-G_{1m-1}{\left(t\right)\|}_{\infty }\;\|G_{2m+1}-G_{2m}\|\;\leq \partial_{6}\|G_{2m}\left(t\right)-G_{2m-1}{\left(t\right)\|}_{\infty }\;\|R_{1m+1}-R_{1m}\|\;\leq \partial_{7}\|R_{1m}\left(t\right)-R_{1m-1}{\left(t\right)\|}_{\infty }\;\|R_{2m+1}-R_{2m}\|\;\leq \partial_{8}\|R_{2m}\left(t\right)-R_{2m-1}{\left(t\right)\|}_{\infty }$$

The above inequalities prove the existence of the function H. We now show the uniqueness of the solution by assuming that the kernels $H_{i},\;i=1,2,\ldots ,\;8$ are separable, i.e., $H_{1}\left(t,\epsilon \right)=\emptyset \left(t\right)\sigma \left(\epsilon \right)$. By denoting:

$$\zeta \left(t\right)=\int_{0}^{t}S_{1}\left(\epsilon \right)\sigma \left(\epsilon \right)d\epsilon$$

then, because $S_{1}\left(t\right)=S_{1}\left(0\right)+\int_{0}^{t}H_{1}\left(t,\epsilon \right)S_{1}\left(\epsilon \right)d\epsilon =S_{1}\left(0\right)+\int_{0}^{t}\emptyset \left(t\right)\sigma \left(\epsilon \right)S_{1}\left(\epsilon \right)d\epsilon ,\;$ we have:

$$\;\zeta \;\prime \left(t\right)=\emptyset \left(t\right)\sigma \left(t\right)\zeta \left(t\right)+{\;S}_{1}\left(0\right)\sigma \left(t\right)$$

If $\sigma \left(0\right)=0$, the solution $\zeta \left(t\right)$ follows $\zeta \left(t\right)={\;e}^{\int_{0}^{t}\emptyset \left(\epsilon \right)\sigma \left(\epsilon \right)d\epsilon }$ and from the definition of $\zeta \left(t\right)$, the solution of $S_{1}\left(t\right)$ is given as:

$$S_{1}\left(t\right)=S_{1}\left(0\right)+\emptyset \left(t\right)\int_{0}^{t}S_{1}\left(\epsilon \right)\sigma \left(\epsilon \right)d\epsilon ={\;S}_{1}\left(0\right)+\emptyset \left(t\right)\zeta \left(t\right)=S_{1}\left(0\right)+\emptyset \left(t\right)e^{\int_{0}^{t}\emptyset \left(\epsilon \right)\sigma \left(\epsilon \right)d\epsilon }$$

Hence, by the unicity of the solution $\zeta \left(t\right)$, there exists just only one continuous solution for $S_{1}\left(t\right)$. Following the same approach, we can obtain a unique solution for the remaining equations of the system (2).

### 4.6. Basic Reproduction Number

In this section, we apply the idea of a next-generation matrix by linearizing the model Equation (1) near the endemic state, for the infectious part of the system (1), and we obtain:

(4) $${\mathrm{dI}}_{1}/\mathrm{dt}=\varphi_{1}\left(I_{1}+I_{2}\right)S_{1}{}^{**}-(a_{1}^{I}+\eta_{1})I_{1}\;,\;{\mathrm{dI}}_{2}/\mathrm{dt}=\varphi_{2}\left(I_{1}+I_{2}\right)S_{2}{}^{**}+a_{1}^{I}I_{1}-\eta_{2}I_{2\;}-\;\mu_{2}{}^{I}I_{2},\;{\mathrm{dG}}_{1}/\mathrm{dt}={\;\eta }_{1}I_{1}+{\;\theta }_{1}^{G}S_{1}{}^{**}\;–\;(a_{1}^{G}+\gamma_{1}+\epsilon_{1})G_{1},\;{\mathrm{dG}}_{2}/\mathrm{dt}={\;\eta }_{2}I_{2}+{\;a}_{1}^{G}G_{1}+{\;\theta }_{2}^{G}S_{2}{}^{**}\;–\;(\gamma_{2}+{\;\mu }_{2}{}^{G}+\epsilon_{2}+\beta_{22})G_{2}$$

By summing Equation (4) and by denoting I as the size of all infectious, we have:

I(t) = I_1_(t) + I_2_(t) + G_1_(t) + G_2_(t), and

dI/dt = $\varphi_{1}\left(I_{1}+I_{2}\right)S_{1}{}^{**}+\varphi_{2}\left(I_{1}+I_{2}\right)S_{2}{}^{**}\;$− $\mu_{2}{}^{I}$ I_2_$+{\;\theta }_{1}^{G}S_{1}{}^{**}-(\gamma_{1}+\epsilon_{1})G_{1\;}-(\gamma_{2}+\epsilon_{2}+\beta_{22})G_{2}$

The matrix J of the linearized system near a state (S_1_, S_2_) is:

$$J=\left[\begin{array}{cccc} \varphi_{1}S_{1}-a_{1}^{I}-\eta_{1} & \varphi_{1}S_{1} & 0 & 0\\ \varphi_{2}S_{2}+a_{1}^{I} & \varphi_{2}S_{2}-\eta_{2}-{\;\mu }_{2}{}^{I} & 0 & 0\\ \eta_{1} & 0 & -a_{1}^{G}-\gamma_{1}-\epsilon_{1} & 0\\ 0 & \eta_{2} & a_{1}^{G} & -\gamma_{2}-{\;\mu }_{2}{}^{G}-\epsilon_{2}-\beta_{22} \end{array}\right]$$

The corresponding characteristic polynomial P_J_(λ) is equal to:

$$(\varphi_{1}S_{1}-a_{1}^{I}-\eta_{1}-\;\lambda )(\varphi_{2}S_{2}-\eta_{2}-{\;\mu }_{2}{}^{I}-\;\lambda )\;\left(-a_{1}^{G}-\gamma_{1}-\epsilon_{1}-\;\lambda \right)\;\left(-{µ}_{2}^{G}-\gamma_{2}-\epsilon_{2}-\beta_{22}-\;\lambda \right)\;-{\;\varphi }_{1}S_{1}\left(\varphi_{2}S_{2}+a_{1}^{I}\right)\left(-a_{1}^{G}-\gamma_{1}-\epsilon_{1}-\;\lambda \right)\left(-{µ}_{2}^{G}-\gamma_{2}-\epsilon_{2}-\beta_{22}-\lambda \right)=\;P_{J}(\lambda )$$

The positive eigenvalues of J are roots of the following polynomial:

$$\lambda^{2}-\left(\varphi_{1}S_{1}-a_{1}^{I}-\eta_{1}+\varphi_{2}S_{2}-\eta_{2}-{\;\mu }_{2}{}^{I}\right)\lambda -{\;\varphi }_{1}S_{1}\left(\varphi_{2}S_{2}+a_{1}^{I}\right)+(\varphi_{1}S_{1}-a_{1}^{I}-\eta_{1})(\varphi_{2}S_{2}-\eta_{2}-{\;\mu }_{2}{}^{I})$$

These roots are equal to: B ± (B^2^ − C)^1/2^, where B and C are equal to:

$$B=\frac{\varphi_{1}S_{1}-a_{1}^{I}-\eta_{1}+\varphi_{2}S_{2}-\eta_{2}-{\;\mu }_{2}{}^{I}}{2}\;C=-{\;\varphi }_{1}S_{1}\left(\varphi_{2}S_{2}+a_{1}^{I}\right)+(\varphi_{1}S_{1}-a_{1}^{I}-\eta_{1})(\varphi_{2}S_{2}-\eta_{2}-{\;\mu }_{2}{}^{I})$$

Then, we have:

$$B+(B^{2}-C{)}^{1/2}=\frac{\varphi_{1}S_{1}-a_{1}^{I}-\eta_{1}+\varphi_{2}S_{2}-\eta_{2}-{\;\mu }_{2}{}^{I}}{2}+[\frac{{\left(\varphi_{1}S_{1}-a_{1}^{I}-\eta_{1}+\varphi_{2}S_{2}-\eta_{2}-{\;\mu }_{2}{}^{I}\right)}^{2}}{4}+{\;\varphi }_{1}S_{1}\left(\varphi_{2}S_{2}+a_{1}^{I}\right)-(\varphi_{1}S_{1}-a_{1}^{I}-\eta_{1})(\varphi_{2}S_{2}-\eta_{2}-{\;\mu }_{2}{}^{I}){]}^{1/2}$$

Hence, near unstable endemic state the positive dominant eigenvalue Λ is equal to:

$$\Lambda =\frac{\varphi_{1}S_{1}-a_{1}^{I}-\eta_{1}+\varphi_{2}S_{2}-\eta_{2}-{\;\mu }_{2}{}^{I}}{2}+{[\frac{{\left(\varphi_{1}S_{1}-a_{1}^{I}-\eta_{1}-\varphi_{2}S_{2}+\eta_{2}+{\;\mu }_{2}{}^{I}\right)}^{2}}{4}+{\;\varphi }_{1}S_{1}\left(\varphi_{2}S_{2}+a_{1}^{I}\right)}^{}{]}^{1/2}$$

Therefore, the basic reproduction number R_0_ equal to Λ near the endemic stationary state depends mainly on the infection rates $\varphi_{1}\;$ and ${\;\varphi }_{2}$, when the sizes S_1_ and S_2_ are sufficiently important. If after a change of parameter values, the endemic state becomes unstable and R_0_ is becoming more than 1, then an epidemic wave starts.

## 5. Numerical Simulation of the Model: Some Examples of the COVID-19 Outbreak in Kuwait, France and Cameroon

First, we provide some explanations for the data used for the simulations, including how we assumed some of the parameters, calculated others, and selected some from the literature cited in this article. In order to determine the susceptible classes sizes, we used Wikipedia data on the three studied countries to obtain their total population size. We then calculated the ratio between the young and elderly from the data presented in Figure 2, Figure 3 and Figure 4 and used this ratio to determine the sizes value for the two susceptible classes at the exponential phase considered (for Kuwait 28 December 2020, France 30 October 2021, and Cameroon 19 September 2021), because the Ross and Kermack-McKendrick model is only suited for the exponential growth phase of an epidemic wave. Because the progression rates to the classes reversed recovery and fully recovered in Figure 5 are not zero, we assumed that they should have some populations at the start of the wave, even if they were small. The transmission rate for Cameroon was chosen at the start of the second wave in January 2021 from [56,57], for France at the start of the fifth wave in December 2021 [58], and for Kuwait at the start of the fourth wave in December 2021 [59]. The values of specific death rates due to disease and natural death rates for Kuwait and France were taken from [58,59,60]. For Cameroon, the natural death rate was taken from [61], while the specific death rate due to disease was calculated from the cumulated deaths number due to the disease in two years divided by the cumulated infectious number in these two years [61]. The vaccination rate was chosen from [62], while the loss of resistance was chosen from [63]. Other parameters were assumed.

### 5.1. COVID-19 Outbreak in Kuwait

We have chosen the following set of parameter values corresponding to the COVID-19 outbreak at the start of the fourth wave in December 2021 in Kuwait:

$$\varphi_{1\;}=1.7,\varphi_{2\;}=0.9,\mu_{2}{}^{S}=0.003,\epsilon_{1\;}=0.28,\beta_{22\;}=0,{\;\mu }_{2}{}^{I}=0.0025,{\;\mu }_{2}{}^{G}=\;0.002,{\;\mu }_{2}{}^{R}=0.0021\;,{\;\beta }_{11\;}=2.1\;,\beta_{21\;}=2.3\;,{\;\theta }_{1}^{G}\;=0.765,\theta_{1}^{R}=0.678,\gamma_{1}=\;0.62,\gamma_{2}=0.74,{\;\theta }_{2}^{G}\;=0.45\;,\theta_{2}^{R}=0.33,{\;a}_{1}^{S}=0.7,a_{1}^{I}=0.54,a_{1}^{G}=0.65,a_{1}^{R}=\;0.8,\;\epsilon_{2\;}=0.19\;,\eta_{1}=0.3{\;\mathrm{and}\;\eta }_{2}=0.38.$$

with initial values for S_1_ = 4413099, S_2_ = 51422, I_1_ = 65, I_2_ = 194, G_1_ = 20, G_2_ = 17, R_1_ = 30 and R_2_ = 73.

**Figure 6:**
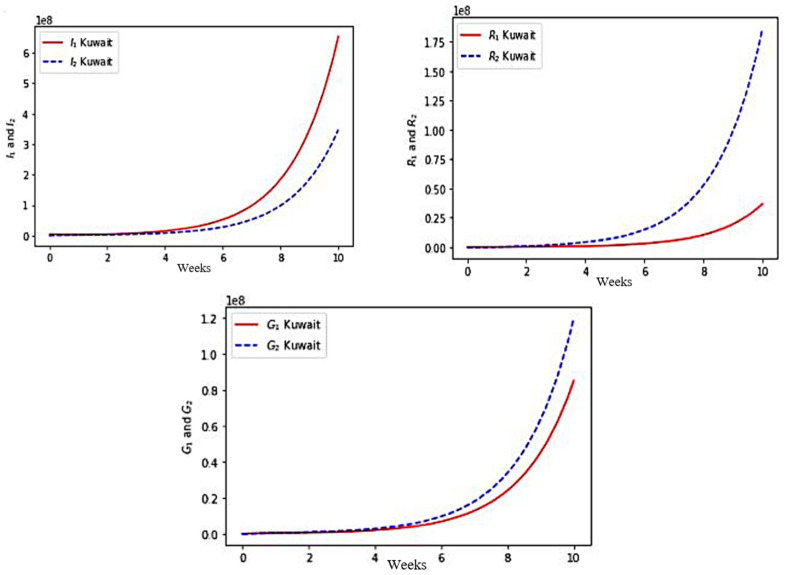
Numerical simulation of the variables I_1_ and I_2_, R_1_ and R_2_, G_1_ and G_2_ for Kuwait.

We present the visualization results for the simulated values in Figure 6.

### 5.2. COVID-19 Outbreak in France

We have chosen the following set of parameter values corresponding to the COVID-19 outbreak at the start of the fifth wave in December 2021 in France:

$$\varphi_{1\;}=\;1.2,\varphi_{2\;}=\;0.9,\mu_{2}{}^{S}=\;0.009,\epsilon_{1\;}=\;0.28,\beta_{22\;}=\;0,{\;\mu }_{2}{}^{I}=\;0.0025,{\;\mu }_{2}{}^{G}=\;0.002,{\;\mu }_{2}{}^{R}=0.0021\;,{\;\beta }_{11\;}=\;1.9,\beta_{21\;}=\;2.2,{\;\theta }_{1}^{G}\;=\;0.735,\theta_{1}^{R}=\;0.678,\gamma_{1}=\;0.62,\gamma_{2}=0.74,{\;\theta }_{2}^{G}\;=0.45,\theta_{2}^{R}=0.33,{\;a}_{1}^{S}=\;0.7,a_{1}^{I}=\;0.54,a_{1}^{G}=\;0.65,a_{1}^{R}=\;0.8,\;\epsilon_{2\;}=\;0.19,\eta_{1}=\;0.3{\;\mathrm{and}\;\eta }_{2}=\;0.38.$$

with initial values for S_1_ = 53372880, S_2_ = 14017120, I_1_ = 3912, I_2_ = 1757, G_1_ = 200, G_2_ = 170, R_1_ = 300 and R_2_ = 730.

**Figure 7:**
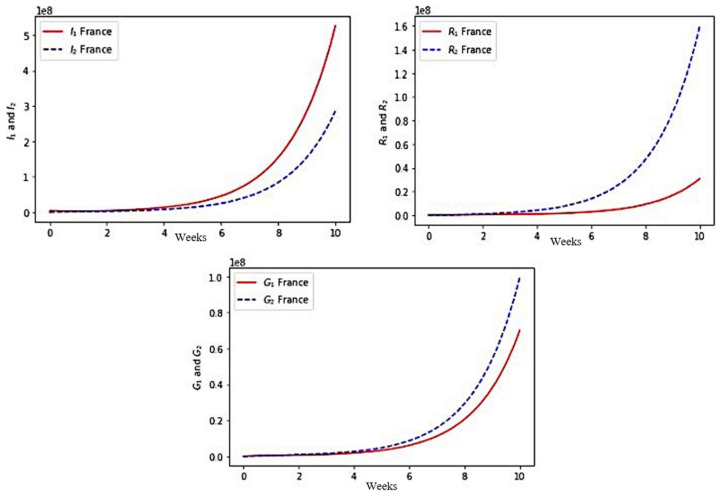
Numerical simulation of the variables I_1_ and I_2_, R_1_ and R_2_, G_1_ and G_2_ for France.

We present the visualization results for the simulated values in Figure 7.

### 5.3. COVID-19 Outbreak in Cameroon

We choose the following set of parameter values corresponding to the COVID-19 outbreak at the start of the second wave in January 2021 in Cameroon:

$$\varphi_{1\;}=1.3,\varphi_{2\;}=0.9,\mu_{2}{}^{S}=0.009,\epsilon_{1\;}=0.28,\beta_{22\;}=0,{\;\mu }_{2}{}^{I}=0.0025,{\;\mu }_{2}{}^{G}=\;0.002,{\;\mu }_{2}{}^{R}=0.0021\;,{\;\beta }_{11\;}=4.5,\beta_{21\;}=4.1,{\;\theta }_{1}^{G}\;=0.024,\theta_{1}^{R}=0.678,\gamma_{1}=\;0.62,\gamma_{2}=0.74,{\;\theta }_{2}^{G}\;=0.45,\;\theta_{2}^{R}=0.33,{\;a}_{1}^{S}=0.7,a_{1}^{I}=0.54,a_{1}^{G}=0.65,a_{1}^{R}=\;0.8,\;\epsilon_{2\;}=0.19,\eta_{1}=0.3{\;\mathrm{and}\;\eta }_{2}=0.38.$$

with initial values for S_1_ = 25828973, S_2_ = 721027, I_1_ = 151, I_2_ = 21, G_1_ = 75, G_2_ = 33, R_1_ = 56 and R_2_ = 64.

**Figure 8:**
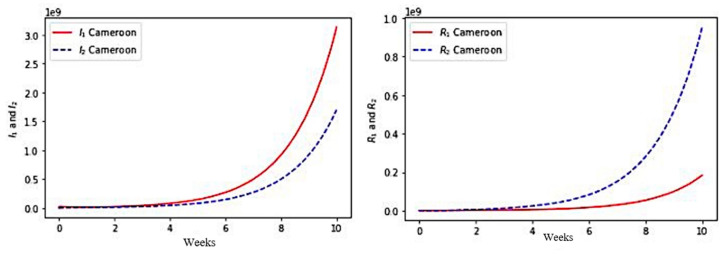

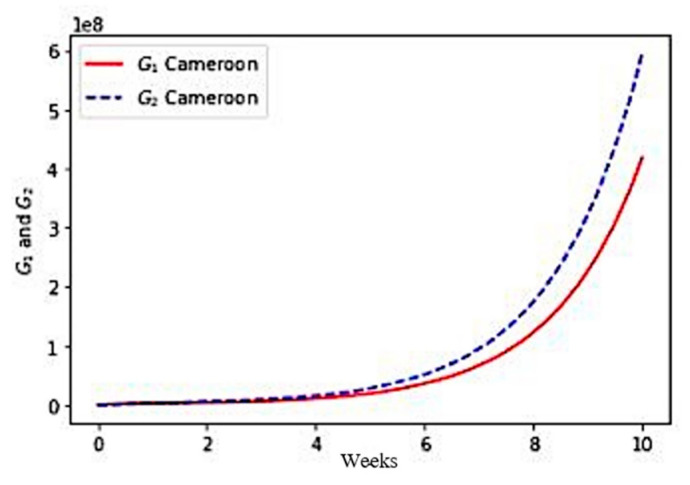
Numerical simulation of the variables I_1_ and I_2_, R_1_ and R_2_, G_1_, G_2_ for Cameroon.

We present the visualization results for the simulated values in Figure 8.

## 6. Discussion

The results for the three considered countries present some similarities but also some differences that we will discuss in the following.

First, in each case, the exponential growth of the infectious I_1_ + I_2_ and completely recovering R_1_ + R_2_ populations sizes correspond roughly to the data given in [57]. The model simulations give more, i.e., allows to see the part brought by each age class to the global growth.

Second, for Kuwait, Figure 6 shows a faster growth of infectious (I) in the young class (≤65 years) than in the older class (>65 years), which is also the case for France (Figure 7) and for Cameroon (Figure 8). On the other hand, this phenomenon is reversed in the three countries, with regard to the growth of populations immunized in a transient manner (G) and in a lasting manner (R). The phenomenon is more marked for Kuwait than for France, France itself having a more marked difference than for Cameroon. This is partly explained by the better vaccination rate of Kuwait than that of France and Cameroon in the young class, the effectiveness of the vaccination (which decreases in the older class) having been assumed to be equal for the three countries.

## 7. Conclusions

Taking age into account in modeling the COVID-19 pandemic makes it possible to simulate the differential dynamic behavior of the growth of infectious and immune populations, young and old, in order, for example, to adjust the vaccine policy according to the age. Future work should, for example, take into account more age groups, at least four: children (age ≤ 12 years), adolescents (12 years < age ≤ 18 years), young adults (18 years < age ≤ 65 years), and older adults (>65 years). The most important pitfall in simulations of such a model is the estimation (by observation, calculation, or assumption) of its parameters, already difficult with two age groups. A random choice of the values of the parameters in plausible intervals followed by a study of the sensitivity to the parameters of the model, could make it possible to partly overcome the constraint of parameter estimation in a more precise future epidemiological–demographic model, in particular with regard to age groups.

Another perspective would consist in introducing, thanks to Usher’s model [41,42], an accelerated aging due to infection, as well as an influence of exogenous determinants such as geo-climatic, socio-economic, and health-related factors [64,65,66,67,68,69,70,71,72,73,74,75,76], which weight differently on the different age groups, therefore, changing the growth dynamics specific to each of the sub-populations studied in this article. These perspectives will be examined in future works.

## Figures and Tables

**Table 1:** List of the parameters considered in the SIGR (Susceptible–Infectious–Goneanewsusceptible–Recovered) model.

Epidemiologic Parameters	
S_i_	Susceptible individuals of age class i among age classes, adult 1 and elderly 2.
I_i_	Infectious individuals of age class i
G_i_	Gone anew susceptible individuals of age class i
R_i_	Fully recovered and resistant individuals of age class i
R_o_	Basic reproduction number
ϕ_i_	Transmission rate of age class i
η_i_	Relapsed rate of age class i
γ_i_	Recovering rate of age class i
a_1_^K^	Survival rate from age 1 to age 2 for compartment K of age class 1
µ_2_^K^	Natural death rate of compartment K of age class 2
**Demographic Parameters**	
β_i1_	Birth and loss of resistance of recovered from compartments of age class i
β_22_	Loss of resistance of recovered rate from compartments of age class 2
θ_i_^K^	Vaccination rate from compartment K of age class i
$\epsilon_{i}$	Specific fatality rate ϵ due to the disease of age class i
